# Comparison of survival outcomes of locally advanced cervical cancer by histopathological types in the surveillance, epidemiology, and end results (SEER) database: a propensity score matching study

**DOI:** 10.1186/s13027-020-00299-3

**Published:** 2020-05-13

**Authors:** Tian Tian, Xing Gong, Xudong Gao, Yanqing Li, Wen Ju, Yiqin Ai

**Affiliations:** 1 Department of Radiation Therapy, The Third Affiliated Hospital of Kunming Medical University, Kunming, 650118 Yunnan China; 2 Department of Radiation Therapy, The Tumor Hospital of Yunnan Province, Kunming, 650118 Yunnan China; 3 Department of Oncology, Xiang Yang NO.1 People’s Hospital, Affiliated Hospital of Hubei University of Medicine, Xiang Yang, 441000 China; 4 519 Kunzhou Road, Kunming, China

**Keywords:** SEER, Squamous cell carcinoma, Adenocarcinoma, Cervical cancer, Propensity score matching

## Abstract

**Background:**

There has been limited research on the comparison of squamous cell carcinoma (SCC) and adenocarcinoma (AC) of cervical cancer and that lack of information may have significant bearing on the treatment of patients. We compared survival outcomes between squamous cell carcinoma and adenocarcinoma in locally advanced cervical cancer patients and examined factors related to the prognosis of cervical cancer.

**Methods:**

We identified 4131 patients with stage IB2-IVA cervical cancer patients diagnosed between 2010 and 2015 by using the Surveillance, Epidemiology, and End Results (SEER) database. Variables related to the prognosis of cervical cancer were compared using both univariate and multivariate Cox models and log-rank method before and after propensity score matching. We compared the efficacy of radiotherapy alone to radiotherapy combined with chemotherapy or/and surgery in overall survival of SCC and AC.

**Results:**

Our sample included 3385 patients with SCC (81.9%) and 746 patients with AC (18.1%). The 5-year overall survival on comparing the squamous cell carcinoma group and adenocarcinoma group was not significant (*P* > 0.05). Using propensity score matching, 676 pairs of patients were selected. The 5-year overall survival of matched patients did not differ significantly (*P* > 0.05). Histology was not independently associated with overall survival in multivariate Cox model (P > 0.05). Factors affecting overall survival included FIGO stage IVA (*P* < 0.05), chemotherapy (P < 0.05), and external radiation combined with brachytherapy (P < 0.05). Patients with SCC that were treated with radiation alone had significantly worse OS than AC patients receiving radiation only (*P* < 0.05).

**Conclusions:**

The OS in AC of the cervix is similar to that SCC in when treated with radiotherapy combined with chemotherapy and/or surgery but better when treated with radiation alone.

## Background

Cervical cancer is one of the common cancers in women worldwide. According to the World Health Organization, the incidence of cervical cancer ranks first in 28 countries and it is the leading cause of female deaths in 42 countries and it is estimated that 570,000 new cases and 311,000 deaths may occur worldwide in 2019 [1]. In China, cervical cancer ranked 8th among the top 10 malignant tumors in 2015, accounting for 2.83% of all cancers. The incidence of cervical cancer in rural areas is higher than that in cities (6.34 and 2.68%, respectively) and the average age of onset of cervical cancer is 51 years, primarily in the 40–50 years age range. Cervical cancer rarely occurs before 20 years of age [2].

Cervical cancer includes squamous cell carcinoma (SCC), adenocarcinoma (AC), adenosquamous carcinoma (ASC), neuroendocrine cancer, undifferentiated cancer, and other pathological types. SCC accounts for approximately 70% and AC accounts for approximately 25% cases [3]. High-risk human papilloma virus (HPV) is a significant risk factor for cervical cancer. At present, more than 100 different subtypes of HPV have been found and 54 types able to infect the reproductive tract mucosa. High-risk subtypes include HPV16, 18, 31, 33, 35, 39, and 45. HPV16 and 18 subtypes are most closely related to cervical cancer [4]. In recent years, HPV vaccines and screenings have reduced the incidence of cervical cancer in developed countries. However, the age-adjusted incidence rates of AC increased by 0.5 to 3% per annum in Europe [5]. It is still controversial as to whether the pathological types of cervical cancer have an impact on the prognosis of patients. Some studies have shown that SCC and AC have the same overall survival (OS) [6, 7], while some data suggest that patients with AC of the cervix had poorer OS and disease-free survival (DFS) than patients with SCC [3, 8–10].

In the current National Comprehensive Cancer Network (NCCN) guidelines, the standard treatment methods revealed no differences between SCC and AC of cervical cancer. However, the prognosis and clinical characteristics of SCC cervical cancer differs from that of AC cervical cancer. It has been reported that patients with AC tend to be younger and Caucasian, and they are more commonly diagnosed in the early stages of disease [11].

Some studies have shown that, when compared with radiotherapy alone, concurrent radiotherapy combined with platinum-based chemotherapy can improve survival [12–17]. Since 1999, concurrent chemoradiotherapy has become the standard treatment approach for locally advanced cervical cancer regardless of the histological subtype of the disease. In recent years, some studies have suggested that total hysterectomy after neoadjuvant chemotherapy can improve survival outcomes of cervical cancer [18, 19]. This may be because neoadjuvant chemotherapy has the potential to eradicate micrometastases and could reduce systemic failures, in addition to facilitating local control by surgical resection. However, other studies have shown that the effect of neoadjuvant chemotherapy plus surgery does not show superiority compared to concurrent chemoradiotherapy [20, 21].

Therefore, we conducted this retrospectively study to analyze the data of patients with cervical cancer in Surveillance, Epidemiology and End Results (SEER) database to compare the survival outcomes of patients with SCC and those with AC among locally advanced cervical cancer patients as well as factors that are related to the prognosis of cervical cancer.

## Methods

### Patients

Information on patients diagnosed with cervical cancer between 2010 and 2015 was extracted from the SEER-18 Regs Custom database using SEER*Stat software, version 8.3.6. We limited our sample to the period 2010–2015, as the information about some variables only are available from 2010. We included cervical cancer patients in our study using the following parameters: only one primary malignancy; International Federation of Gynecology and Obstetrics (FIGO,2009 version) stages IB2 to IVA; pathological biopsy confirmed SCC and AC; no distant metastases; aged 20 to 69 years; active follow-up with complete date; and complete clinicopathological information. Since the patient information in the SEER database is deidentified and publicly available, our study was exempt from Institutional Review Board approval. This study was conducted in concordance with the Helsinki Declaration.

Variables for each patient included: race, age, TNM stage, histology grade, T stage, N stage, pathological subtype, treatment strategy, survival time, and vital status. We restaged TNM stage according to the FIGO classification (2009 version). American Joint Committee on Cancer 7th edition (AJCC7) provided updated guidelines on the grading of cervical cancer. Patients were treated either with radiation alone, radiotherapy combined with chemotherapy, and/or surgery. Radiotherapy primarily included external radiation, brachytherapy, or external radiation combined with brachytherapy. The primary endpoint of this study was OS.

### Statistics analyses

The baseline characteristics between cervical cancer patients in the AC and SCC groups were compared with chi-square (χ^2^) or continuity correction tests. A Cox proportional hazards model was used to perform univariate and multivariate analyses of the various factors affecting OS. As the baseline characteristics were different between two groups, propensity score matching (PSM) was performed with a ratio of 1:1. The matching covariates included race, age, TNM stage, histology grade, radiation sequence with surgery, radiotherapy and chemotherapy. Comparisons between the SCC and AC groups was evaluated by a two-side log-rank test before and after matching and OS was estimated using the Kaplan-Meier method. All analyses were performed using R statistical software (version 3.6.1, package includes survminer, rms, foreign, tableone, broom, matchlt, R Foundation) for statistical analysis. Hazard ratio (HR) and 95% confidence intervals (CIs) were compared between two groups. Two-sided *p* < 0.05 was defined as statistically significant.

## Results and conclusions

### Patient baseline characteristics

There were 4131 eligible patients who were diagnosed with stage IB2 to IVA cervical cancer between 2010 to 2015, including 3385 (81.9%) patients in the SCC group and 746 (18.1%) patients in the AC group. The detailed characteristics of patients in the SCC and AC groups before and after propensity score matching are shown in Table 1. There were significantly more patients between the ages 40 and 49 in the AC group than in the SCC group (28.5% in the SCC group and 33.9% in the AC group, *P* = 0.02). The SCC and AC groups differed significantly when comparing the distribution of well differentiated (grade I) and undifferentiated (grade IV) cancer (4.2 and 1.5% in SCC group, 18.5 and 4.0% in AC group, *P* < 0.001). There were more patients with FIGO stages IIB - IVA in the SCC group (82.8% in SCC group, 71.9% in AC group, P < 0.001). The median survival time of patients in the SCC and AC groups was 33.6 (range, 0–83 months) and 35.8 months (range, 0–83 months), respectively (*P* = 0.013).

**Table 1:** Characteristics of cervical cancer patients with AC and SCC before and after matching

Characteristics	Before matching			After matching		
	AC (*n* = 746)	SCC (*n* = 3385)	*P* value	AC(n = 676)	SCC(*n* = 676)	P value
age (%)			0.02			0.872
< 40	158 (21.2)	840 (24.8)		146 (21.6)	143 (21.2)	
40–49	253 (33.9)	965 (28.5)		222 (32.8)	236 (34.9)	
50–59	199 (26.7)	947 (28.0)		183 (27.1)	179 (26.5)	
60–69	136 (18.2)	633 (18.7)		125 (18.5)	118 (17.5)	
race (%)			< 0.001			0.763
Black	59 (7.9)	568 (16.8)		58 (8.6)	62 (9.2)	
White	596 (79.9)	2476 (73.1)		538 (79.6)	527 (78.0)	
Other	91 (12.2)	341 (10.1)		80 (11.8)	87 (12.9)	
grade (%)			< 0.001			0.928
I	138 (18.5)	142 (4.2)		80 (11.8)	77 (11.4)	
II	313 (42.0)	1610 (47.6)		306 (45.3)	297 (43.9)	
III	265 (35.5)	1583 (46.8)		264 (39.1)	276 (40.8)	
IV	30 (4.0)	50 (1.5)		26 (3.8)	26 (3.8)	
FIGO stage (%)			< 0.001			0.923
Ib2	142 (19.0)	366 (10.8)		118 (17.5)	121 (17.9)	
IIa1	25 (3.4)	81 (2.4)		21 (3.1)	14 (2.1)	
IIa2	42 (5.6)	135 (4.0)		35 (5.2)	38 (5.6)	
IIb	160 (21.4)	732 (21.6)		142 (21.0)	143 (21.2)	
IIIa	15 (2.0)	75 (2.2)		14 (2.1)	11 (1.6)	
IIIb	346 (46.4)	1837 (54.3)		331 (49.0)	333 (49.3)	
IVa	16 (2.1)	159 (4.7)		15 (2.2)	16 (2.4)	
sequence (%)			< 0.001			0.974
No surgery	318 (42.6)	2011 (59.4)		308 (45.6)	302 (44.7)	
RPTS	55 (7.4)	133 (3.9)		45 (6.7)	48 (7.1)	
RAS	365 (48.9)	1218 (36.0)		315 (46.6)	317 (46.9)	
RBAS	8 (1.1)	23 (0.7)		8 (1.2)	9 (1.3)	
Radiation (%)			0.527			0.25
Beam radiation	328 (44.0)	1487 (43.9)		294 (43.5)	323 (47.8)	
brachytherapy	82 (11.0)	328 (9.7)		70 (10.4)	70 (10.4)	
BRB	336 (45.0)	1570 (46.4)		312 (46.2)	283 (41.9)	
Chemotherapy (%)			0.017			0.865
Yes	652(87.4)	3060(90.4)		596(88.2)	599(88.6)	
No	94 (12.6)	325 (9.6)		80 (11.8)	77 (11.4)	
status (%)			0.055			
alive	535 (71.7)	2303 (68.0)		480 (71.0)	486 (71.9)	0.763
dead	211(28.3)	1082(32.0)		196(29.0)	190(28.1)	
time (mean (SD))	35.8(21.4)	33.6(21.7)	0.013	35.0(21.1)	33.7 (21.5)	0.269

AC adenocarcinoma, SCC Squamous cell carcinoma, FIGO the International Federation of Gynecology and Obstetrics, RPTS Radiation prior to surgery, RAS Radiation after surgery, RBAS Radiation before and after surgery, BRB Combination of beam with brachytherapy. Grade I Well differentiated,gradeII Moderately differentiated,gradeIII Poorly differentiated,gradeIV Undifferentiated;SD Standard Deviation

### Survivals of patients in SCC and AC groups

**Fig. 1:**
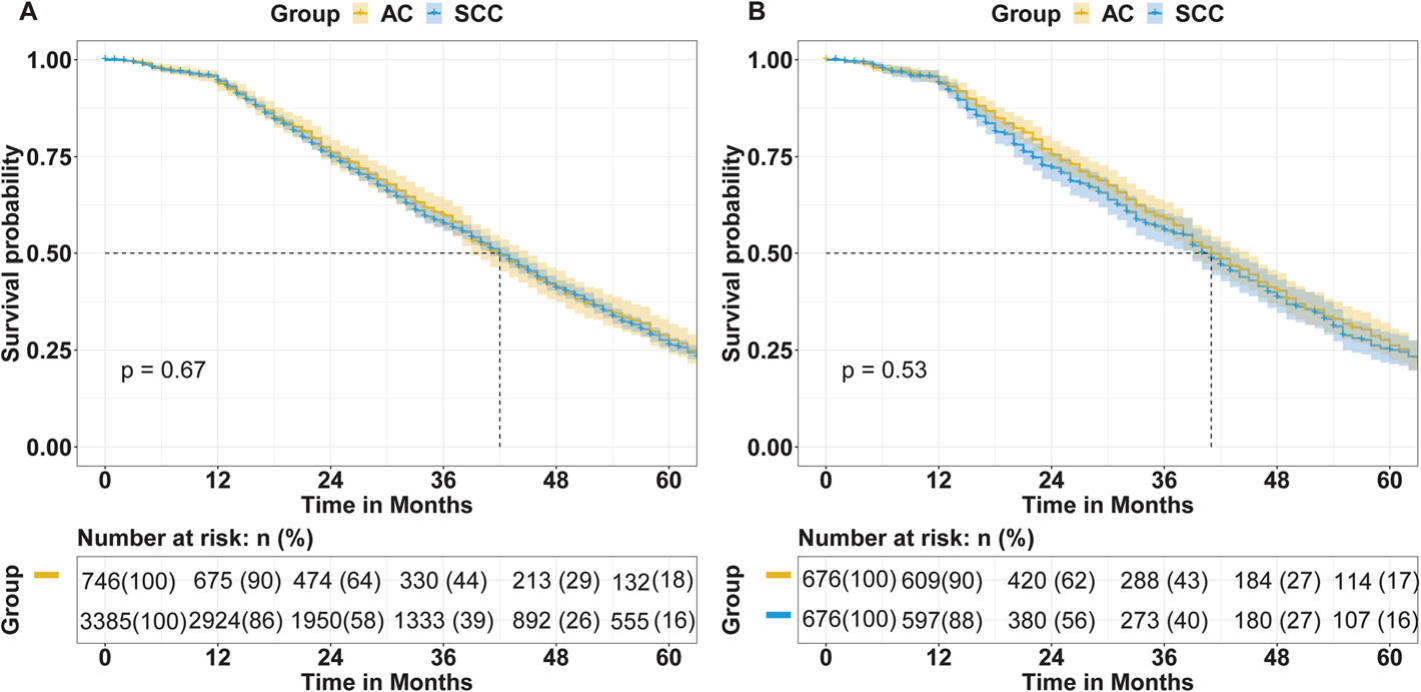
The overall survival (OS) of patients with squamous cell carcinoma (SCC) and adenocarcinoma (AC) (A) before propensity score matching (PSM) (B) after PSM

To balance the baseline characteristics, 676 cervical patients with AC were matched 1:1 to patients with SCC. The baseline characteristics were similar between two groups after matching (Table 1). The OS curves of patients with SCC and AC are shown in Fig. 1. The 5-year OS of patients in the SCC and AC groups were 16 and 18% (*P* = 0.67) before matching and were 16 and 17% after matching (*P* = 0.53).

### Univariate and multivariate analysis

**Fig. 2:**
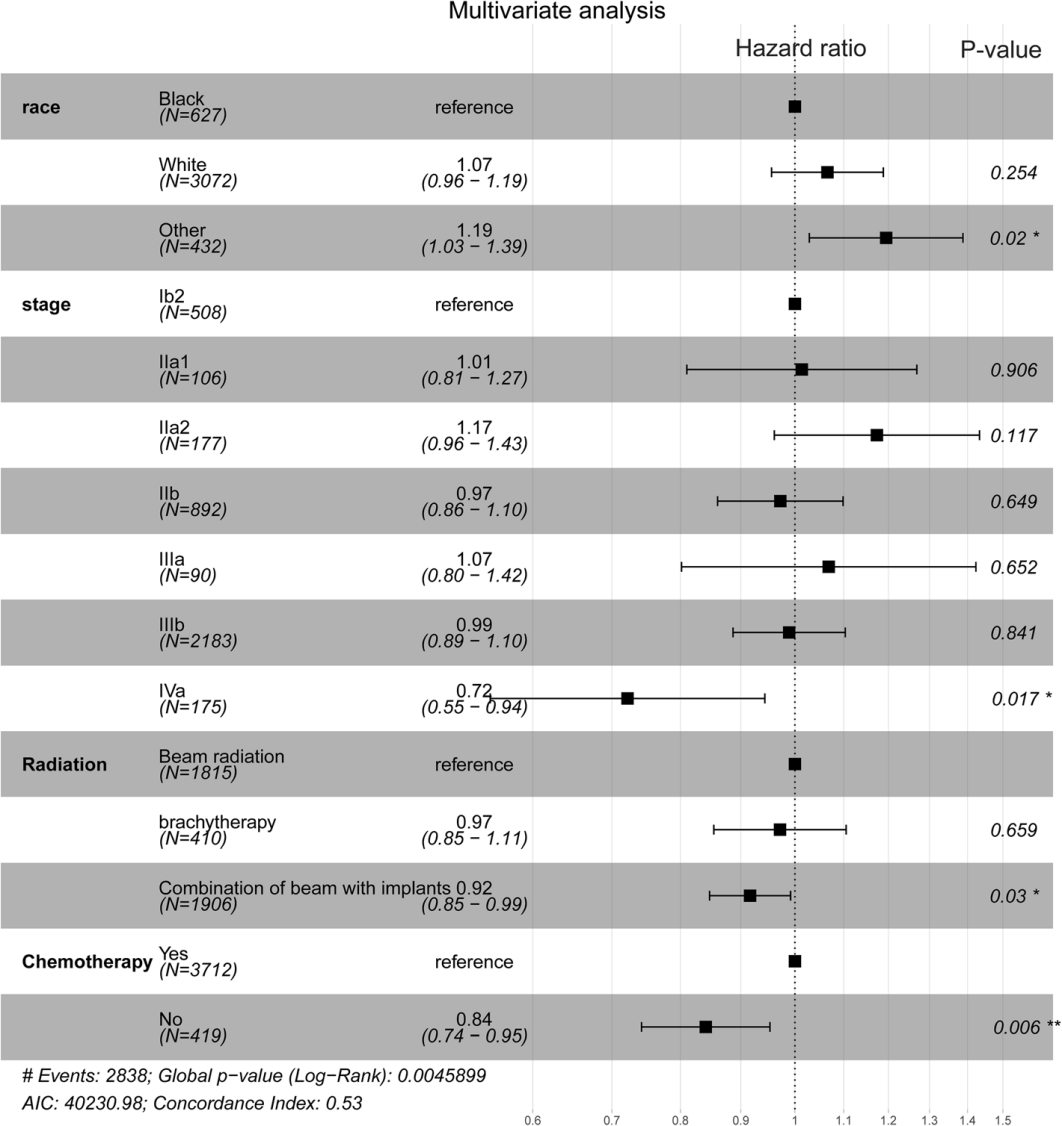
Multivariate analysis of factors associated with OS

In Table 2, we present the results of the univariate analysis showing that histology was not a significant factor for OS. The univariate COX hazard ratio (HR) was 1.02 (95% confidence interval (CI) 0.93–1.12, *P* = 0.69). As shown in Table 2, the primary univariate factors affecting OS were: FIGO stage IVA (HR = 0.74, 95% CI 0.57–0.98, *p* = 0.03), chemotherapy (HR 0.85, 95% CI 0.75–0.97, *p* = 0.01), race (HR = 1.20, 95% CI 1.03–1.39, *p* = 0.02) and external radiation combined with brachytherapy (HR 0.93, 95% CI 0.86–1.00, *p* = 0.06). Multivariate analysis found the same associated factors (Fig. 2).

**Table 2:** Univariate analysis of factors associated with OS

Variables	Univariate (OS)	
	HR (95%CI)	P value
histology		
AC	1.00 (reference)	
SCC	1.02 (0.93–1.12)	0.69
age (%)		
< 40	1.00 (reference)	
40–49	1.03 (0.93–1.13)	0.61
50–59	0.97 (0.88–1.08)	0.59
60–69	1.01 (0.90–1.14)	0.81
race (%)		
Black	1.00 (reference)	
White	1.06 (0.95–1.18)	0.28
Other	1.20 (1.03–1.39)	0.02
grade (%)		
I	1.00 (reference)	
II	1.07 (0.93–1.24)	0.36
III	1.00 (0.86–1.15)	0.95
IV	1.12 (0.82–1.53)	0.47
FIGO stage (%)		
Ib2	1.00 (reference)	
IIa1	1.02 (0.81–1.27)	0.88
IIa2	1.17 (0.96–1.43)	0.12
IIb	0.98 (0.87–1.10)	0.72
IIIa	1.07 (0.80–1.42)	0.64
IIIb	1.00 (0.90–1.11)	0.94
IVa	0.74 (0.57–0.98)	0.03
sequence (%)		
No surgery	1.00 (reference)	
RPTS	0.88 (0.73–1.05)	0.16
RAS	0.93 (0.87–1.01)	0.10
RBAS	1.11 (0.72–1.73)	0.63
Radiation (%)		
Beam radiation	1.00 (reference)	
brachytherapy	0.98 (0.86–1.11)	0.70
BRB	0.93 (0.86–1.00)	0.06
Chemotherapy		
Yes	1.00 (reference)	
No	0.85 (0.75–0.97)	0.01

AC adenocarcinoma, SCC Squamous cell carcinoma, FIGO the International Federation of Gynecology and Obstetrics, RPTS Radiation prior to surgery, RAS Radiation after surgery, RBAS Radiation before and after surgery, BRB Combination of beam with brachytherapy, grade I Well differentiated,gradeII Moderately differentiated,gradeIII Poorly differentiated,gradeIV Undifferentiated;CI indicates confidence interval, HR hazard ratio

### Subgroup analysis

**Fig. 3:**
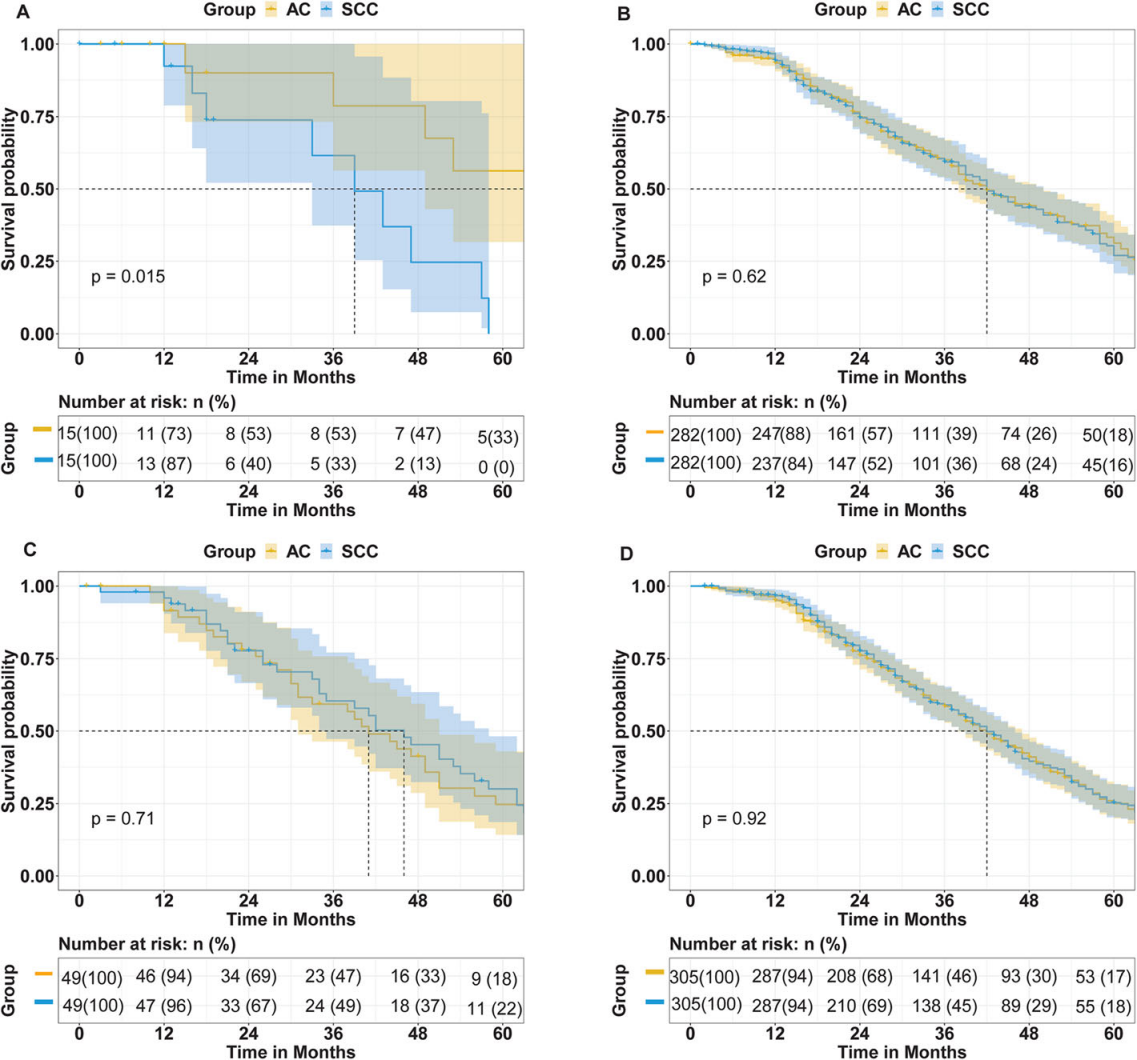
Treated with radiotherapy alone the overall survival (OS) of patients with squamous cell carcinoma (SCC) and adenocarcinoma (AC) (A); treated with radiotherapy and chemotherapy the OS of patients with SCC and AC(B), treated with radiotherapy and surgery the OS of patients with SCC and AC(C); treated with radiotherapy, surgery and chemotherapy the OS of patients with SCC and AC(D)

For these analyses, we stratified patients by treatment and performed 1:1 matching on pathology. The detailed characteristics of subgroups after propensity score matching are shown in Table 3 and Table 4 .After matching, 15 cervical cancer patients with AC were matched with 15 patients with SCC (Fig. 3a) in the radiation only group. We showed that the OS of patients with SCC was significantly worse than that of patients with AC (*P* = 0.015). In the radiation combined with chemotherapy subgroup, we defined 282 matched pairs. The OS of patients receiving radiation combined with chemotherapy did not differ significantly by pathology (*P* = 0.62) (Fig. 3b). Figure 3c shows the OS comparing 49 matched subgroups treated with radiotherapy combined with surgery. Within this subgroup, the median survival time was shorter for AC patients, although these results were not statistically significant. Finally, we analyzed radiotherapy combined with chemotherapy plus surgery in 305 pairs and found that the OS of SCC and AC was very similar (*P* = 0.92) (Fig. 3d).

**Table 3:** characteristics of radiotherapy alone group and radiotherapy combined with chemotherapy group after matching

Characteristics	Radiotherapy alone			CTRT		
	AC (*n* = 15)	SCC(n = 15)	P value	AC(*n* = 282)	SCC(n = 282)	P value
age (%)			0.833			0.901
< 40	2 (13.3)	2 (13.3)		41 (14.5)	41 (14.5)	
40–49	5 (33.3)	6 (40.1)		98 (34.8)	100 (35.5)	
50–59	4(26.7)	2 (13.3)		79 (28.0)	84 (29.8)	
60–69	4(26.7)	5 (33.3)		64 (22.7)	57 (20.2)	
race (%)			0.648			0.969
Black	2 (13.3)	4(26.7)		27 (9.6)	27 (9.6)	
White	13 (86.7)	11 (73.3)		36 (12.8)	38 (13.5)	
Other	0 (0.0)	0 (0.0)		219 (77.6)	217 (76.9)	
grade (%)			1.000			0.991
I	0 (0.0)	0 (0.0)		33 (11.7)	34 (12.1)	
II	4(26.7)	5 (33.3)		116 (41.1)	114 (40.4)	
III	11 (73.3)	10 (66.7)		124 (44.0)	126 (44.7)	
IV	0 (0.0)	0 (0.0)		9 (3.2)	8 (2.8)	
FIGO stage (%)			0.970			0.012
Ib2	2 (13.3)	1 (6.7)		45 (16.0)	25 (8.9)	
IIa1	1 (6.7)	1 (6.7)		5 (1.8)	5 (1.8)	
IIa2	0 (0.0)	1 (6.7)		11 (3.9)	7 (2.5)	
IIb	2 (13.3)	2 (13.3)		77 (27.3)	68 (24.1)	
IIIa	1 (6.7)	1 (6.7)		10 (3.5)	9 (3.2)	
IIIb	7 (46.7)	7 (46.7)		127 (45.0)	146 (51.7)	
IVa	2 (13.3)	2 (13.3)		7 (2.5)	22 (7.8)	
Radiation (%)			0.766			0.545
Beam radiation	11 (73.3)	11 (73.3)		100 (35.5)	91 (32.3)	
brachytherapy	2 (13.3)	3 (20.0)		146 (51.7)	159 (56.4)	
BRB	2 (13.3)	1 (6.7)		36 (12.8)	32 (11.3)	
status (%)			1.000			0.542
alive	9 (60.0)	9 (60.0)		181 (64.2)	173 (61.3)	
dead	6(40.0)	6(40.0)		101(35.8)	109(38.7)	
time(mean(SD))	40.4 (32.5)	26.1(18.6)	0.150	33.5 (21.6)	31.8 (22.5)	0.353

AC adenocarcinoma, SCC Squamous cell carcinoma, FIGO the International Federation of Gynecology and Obstetrics, BRB Combination of beam with brachytherapy. CTRT radiotherapy combined with chemotherapy, grade I Well differentiated,gradeII Moderately differentiated,gradeIII Poorly differentiated,gradeIV Undifferentiated;SD Standard Deviation

**Table 4:** characteristics of radiotherapy plus surgery group and radiotherapy combined with chemotherapy and surgery group after matching

Characteristics	radiotherapy plus surgery			CTRT plus surgery		
	AC(*n* = 49)	SCC(n = 49)	P value	AC(*n* = 305)	SCC(n = 305)	P value
age (%)			0.750			0.291
< 40	9 (18.4)	9 (18.4)		84 (27.5)	78 (25.6)	
40–49	17 (34.7)	14 (28.6)		101 (33.1)	93 (30.5)	
50–59	16 (32.7)	15 (30.6)		74 (24.3)	95 (31.1)	
60–69	7 (14.3)	11 (22.4)		46 (15.1)	39 (12.8)	
race (%)			1.000			0.56
Black	3 (6.1)	3 (6.1)		17 (5.6)	16 (5.2)	
White	2 (4.1)	2 (4.1)		37 (12.1)	29 (9.5)	
Other	44 (89.8)	44 (89.8)		251 (82.3)	260 (85.2)	
grade (%)			0.502			0.927
I	1 (2.0)	1 (2.0)		33 (10.8)	35 (11.5)	
II	30 (61.2)	25 (51.0)		156 (51.1)	153 (50.2)	
III	17 (34.7)	23 (46.9)		109 (35.7)	112 (36.7)	
IV	1 (2.0)	0 (0.0)		7 (2.3)	5 (1.6)	
FIGO stage (%)			0.378			0.066
Ib2	18 (36.7)	17 (34.7)		54 (17.7)	34 (11.1)	
IIa1	3 (6.1)	0 (0.0)		11 (3.6)	7 (2.3)	
IIa2	4 (8.2)	3 (6.1)		19 (6.2)	9 (3.0)	
IIb	5 (10.2)	12 (24.5)		57 (18.7)	60 (19.7)	
IIIa	1 (2.0)	1 (2.0)		2 (0.7)	2 (0.7)	
IIIb	16 (32.7)	15 (30.6)		159 (52.1)	190 (62.3)	
IVa	2 (4.1)	1 (2.0)		3 (1.0)	3 (1.0)	
Radiation (%)			0.659			0.988
Beam radiation	34 (69.4)	34 (69.4)		145 (47.5)	144 (47.2)	
brachytherapy	11 (22.4)	13 (26.5)		136 (44.6)	136 (44.6)	
BRB	4 (8.2)	2 (4.1)		24 (7.9)	25 (8.2)	
status (%)			1.000			
alive	40 (81.6)	40 (81.6)		234 (76.7)	236 (77.4)	0.923
dead	9(18.4)	9(18.4)		71(23.3)	69(22.6)	
time (mean (SD))	37.9 (22.0)	39.4(22.1)	0.749	37.3 (20.6)	37.4(21.0)	0.939

AC adenocarcinoma, SCC Squamous cell carcinoma, FIGO the International Federation of Gynecology and Obstetrics, BRB Combination of beam with brachytherapy. CTRT radiotherapy combined with chemotherapy, grade I Well differentiated,gradeII Moderately differentiated,gradeIII Poorly differentiated,gradeIV Undifferentiated;SD Standard Deviation

## Discussion

Adenocarcinoma is a small pathological subtype in cervical cancer. In recent years, while the incidence of cervical cancer has generally declined [22], the incidence of AC has increased [5]. However, using the same treatment as for SCC may not be appropriate for AC patients [23]. According to NCCN guidelines, the standard treatment for locally advanced cervical cancer is radiotherapy and concomitant chemotherapy using cisplatin, a protocol that was developed in SCC patients. As a result, there has been increasing interest in determining optimum treatments for AC. There are still considerable debates about the prognostic patterns between patients of AC and SCC as well as the proper treatment based on subgroup. ASC and AC cancers together represented 21.3, 22.9 and 24.1% of cases for the years 1988–1993, 1994–1999 and 2000–2005, respectively [10]. A study based on the SEER database by Vijaya Galic, et al. showed that the incidence of AC and ASC was increasing 1–2% every 6 years [9].

With this increasing pattern, researchers are increasingly interested in cervical AC. The literature shows that there is still a significant debate regarding the prognosis of patients with AC and SCC of the cervix. Some studies have shown that SCC of the cervix has a similar OS as that of AC [6]. Other studies have shown that AC is less sensitive to radiotherapy than SCC of the cervix, especially in patients with poor prognostic factors [8]. Therefore, in this study, we collected cervical cancer data from the SEER database to identify predictive factors associated with cervical cancer, and survival outcomes in patients with AC and SCC.

In 2011, a previous study by Katanyoo, et al. included a total of 423 patients with stages IIB–IVA and showed that the 5-year OS rates of AC and SCC were 59.9 and 61.7% (*p* = 0.191), respectively. When all prognostic factors were adjusted, clinical staging was the only factor that influenced OS [6]. In 2014, Rose, et al. performed a retrospective analysis of 1671 patients with cervical cancer and suggested that when treated with radiotherapy alone, the OS of AC was worse than that of SCC. When the treatment was radiotherapy and concomitant platinum-based chemotherapy, the OS of patients is similar [11]. In our study, we saw similar patterns. However, in 2018, Hu, et al. compared the treatment outcomes between SCC and AC of the cervix after definitive radiotherapy or concurrent chemoradiotherapy and showed that, when compared with SCC of the cervix, AC affected younger women, was more aggressive, and had more para-aortic metastatic lymph nodes. The 3-year disease-free survival, OS, local control rate, and distant control rate were worse for AC when compared to SCC [3]. In the same year, a propensity score matching study by Yin, et al. enrolled 181 locally advanced cervical cancer patients who were treated with intensity modulated radiotherapy/volumetric modulated arc therapy and concurrent chemotherapy. The results showed that after a 1:1 ratio PSM, the 5-year OS, DFS, locoregional failure-free survival, and distant metastasis-free survival in the locally advanced cervical cancer of AC were 46.0, 43.3, 70.0, and 45.4%, respectively. These results were significantly lower than the corresponding rates of 90.0, 75.8, 96.6, and 78.8% in the matched locally advanced cervical cancer patients of SCC, respectively (*p* < 0.05) [24]. These studies showed a lower OS of AC in cervical cancer.

However, in the present study, we found that histology is not an independent factor of OS. Before PSM, the 3.5-year OS rates in the AC group were 44 and 18%, and the rates were 39 and 16% in the SCC group, respectively (*p* = 0.67). After PSM, the 3-year and 5-year OS of the AC group was 43 and 17%, compared to 40 and 16% in the SCC group (*P* = 0.53). There was no significant difference in OS between AC group and SCC group, regardless of matching status, leading to different conclusions from the previous studies. When comparing these studies, we did not exclude the impact of different treatments on the prognosis of the two pathological types. To make the results stronger, we divided the treatments into four groups and analyzed the difference between the AC and SCC groups.

In the subgroup analysis, when treated with radiotherapy alone, we found that the OS of patients with SCC was worse than that of patients with AC (*P* = 0.015), in contrast with other studies. The number of people in the analysis was small and the power to detect the difference between AC and SCC may have been too low. When we compared the treated with radiotherapy combined with chemotherapy or radiotherapy combined with chemotherapy plus surgery, the OS of patients with SCC and AC was similar (*P* = 0.62, *P* = 0.92). When treated with radiotherapy combined with surgery, the median survival time and the 3-year and 5-year OS of patients with AC was worse than patients with SCC. However there was no significant difference between two groups (*P* = 0.71). In univariable and multivariable analysis, the factors which had the strongest association with survival outcomes was FIGO stage, chemotherapy and external radiation combined with brachytherapy. The difference of study period, number of patients, criteria of enrolled patients, statistical methods and treatment modalities may help us understand the difference of our results when compared to the literature.

This study has some certain limitations. First, we used retrospective studies and we may have unintentional selectivity bias. Second, because the SEER database only recorded the sequence of radiotherapy and surgery, it did not record the sequence of surgery and chemotherapy, and we could not judge whether neoadjuvant therapy or adjuvant therapy is used in the treatment of cervical cancer patients. There was no recorded objective response time and disease-free survival time after treatment; therefore, we could not compare the difference between SCC and AC. In the future, we may need prospective clinical studies to confirm our conclusions.

## Conclusions

Compared with SCC of the cervix, AC has similar OS when treated with radiotherapy combined with chemotherapy and/or surgery but with better OS when treated with radiation alone. Histology is not an independent factor of OS. The factors affecting the prognosis of cervical cancer are the FIGO stage, chemotherapy, race and external radiation combined with brachytherapy.

## Data Availability

The datasets generated and/or analysed during the current study are available in the Surveillance, Epidemiology, and End Results (SEER) database repository, https://seer.cancer.gov.
